# HBHA-ECSIT interaction disrupts macrophage autophagy to promote Mycobacterium tuberculosis persistence

**DOI:** 10.3389/fimmu.2025.1596568

**Published:** 2025-10-24

**Authors:** Yongqiang Li, Xiuping Jia, Xiaoying Wang, Huilian Qiao, Yueyun Ma

**Affiliations:** ^1^ Department of Clinical Laboratory, Air Force Medical Center, Beijing, China; ^2^ College of Life Science, Northwest University, Xi’an, China

**Keywords:** HBHA, ECSIT, macrophage autophagy, tuberculosis, host-pathogen interaction

## Abstract

Tuberculosis (TB), caused by Mycobacterium tuberculosis (Mtb), remains one of the most significant global health challenges exacerbated by latent tuberculosis infection (LTBI). Heparin-binding hemagglutinin (HBHA), a virulence factor of Mtb, plays a critical role in LTBI by inhibiting autophagy in macrophages, though the underlying molecular mechanism has remained unclear. In this study, we identified the evolutionarily conserved signaling intermediate in Toll pathways (ECSIT) as a direct target of HBHA. Our experiments demonstrated that HBHA binds to ECSIT, disrupting the ECSIT-TRAF6 complex and inhibiting ECSIT ubiquitination in BCG-infected macrophages. Through genetic ablation studies in RAW264.7 macrophages, we found that ECSIT is indispensable for HBHA-mediated autophagy suppression, as evidenced by unchanged LC3-II conversion and Beclin-1 expression in ECSIT-knockdown RAW264.7 following HBHA treatment. Additionally, HBHA significantly enhanced intracellular mycobacterial survival in wild-type but not ECSIT-deficient macrophages, establishing ECSIT as an essential molecular nexus for HBHA-mediated bacterial persistence. Our findings reveal a novel mechanism by which Mtb exploits host ECSIT through HBHA to evade autophagic clearance, thereby promoting bacterial persistence. This study identifies the HBHA-ECSIT axis as a potential therapeutic target for host-directed interventions against tuberculosis.

## 1 Introduction

Tuberculosis (TB), caused by Mycobacterium tuberculosis (Mtb), is a chronic infectious disease that remains one of the most significant global health challenges (1, 2). According to the World Health Organization’s Global Tuberculosis Report 2024, there were approximately 8.2 million new TB cases and 1.25 million deaths in 2023 (3). Notably, nearly 25% of the global population harbor latent tuberculosis infection (LTBI), forming a vast reservoir of asymptomatic carriers, and 5-10% of LTBI carriers will progress to active TB during their lifetime (4–6). Furthermore, LTBI serves as a reservoir contributing to drug-resistant tuberculosis emergence (7, 8) and the annual number of people who developed multidrug-resistant TB (MDR-TB) and rifampicin-resistant TB (RR-TB) between 2020–2023 was consistently estimated at approximately 400,000 (2, 3, 9). Elucidating the molecular mechanisms underlying Mtb persistence in hosts and identifying critical host-pathogen interaction nodes are therefore essential for preventing latent reactivation and curbing drug resistance.

The pathogenesis of tuberculosis is intricately related to macrophage autophagy (10). Mtb is an intracellular pathogen mainly transmitted to the lungs through inhalation of aerosolized droplets harboring TB bacteria (11). As the primary host cells targeted by Mtb, macrophages employ autophagy, a conserved self-degradation mechanism in eukaryotic cells for eliminating misfolded proteins, damaged organelles, and pathogenic organisms, as a crucial defense mechanism against intracellular pathogens (12–15). However, autophagy in macrophages infected with Mtb is significantly inhibited. Mtb proteins CpsA and PPE2 would block NADPH oxidase on mycobacterial phagosomes to impair LC3-associated phagocytosis (16, 17). Our previous research identified that the heparin-binding hemagglutinin (HBHA), encoded by the Mtb gene Rv0475, is a dormancy-associated Mtb virulence factor, potently inhibits macrophage autophagy to facilitate bacterial survival (18–20). This autophagy suppression correlates strongly with LTBI establishment, yet its molecular mechanisms remain elusive.

Through HuProt™ human proteome microarray screening, we identified an interaction between HBHA and the evolutionarily conserved signaling intermediate in Toll pathways (ECSIT), a crucial component of innate immunity. ECSIT orchestrates TLR/NF-κB signaling via TRAF6 (TNF receptor-associated factor 6) coupling and serves as a substrate for Parkin-mediated mitophagy, positioning it as a molecular nexus linking immune activation to autophagy regulation (21–23).

Based on these findings, we hypothesize that HBHA disrupts ECSIT ubiquitination networks to inhibit autophagy and promote Mtb latency. To prove this, we utilized ECSIT-knockdown macrophages to systematically elucidate how HBHA-ECSIT interaction modulates autophagic flux. This study reveals a novel mechanism of pathogen-mediated host protein hijacking during LTBI, providing a mechanistic foundation for developing targeted therapies against persistent Mtb infections.

## 2 Materials and methods

### 2.1 Reagents and antibodies

Bacterial culture media including 7H9 Middlebrook broth and 7H10 Middlebrook agar supplemented with oleic acid-albumin-dextrose-catalase (OADC) were purchased from BD Biosciences (San Diego, CA, USA). Tween 80 and glycerol were obtained from Sigma-Aldrich (St. Louis, MO, USA). RPMI1640 medium, fetal bovine serum (FBS) and Penicillin-Streptomycin Liquid were purchased from Gibco (Waltham, MA, USA). Primary antibodies against LC3, beclin1 and GAPDH were purchased from Abcam (Cambridge, MA, USA). FITC-conjugated goat anti-rabbit IgG (H+L) secondary antibody and 180kd protein molecular weight markers were obtained from Beyotime Biotechnology (Shanghai, China).

### 2.2 Bacteria strains and cell lines

The recombinant HBHA-expressing Mycobacterium smegmatis (MS) strain mc^2^155(rHBHA-MS) was constructed in our previous study (18). Mycobacterium bovis bacillus Calmette-Guérin (BCG) was obtained from ATCC (No.35734). rHBHA-MS and BCG were grown in 7H9 Middlebrook broth supplemented with 10% oleic acid-albumin-dextrose-catalase (OADC), 0.05% Tween 80 and 0.2% glycerol, or on 7H10 Middlebrook agar supplemented with 10% OADC. The murine macrophage cell line RAW264.7 was purchased from Procell Life Science & Technology Co., Ltd. (Wuhan, China). ECSIT knockdown RAW264.7 (ECSIT^KD^ RAW264.7) was generated by transduction of shRNA in our previous research. RAW264.7 and ECSIT^KD^ RAW264.7 were cultured in RPMI1640 medium containing 10% fetal bovine serum, 100 units/mL penicillin, and 100 μg/mL streptomycin.

### 2.3 HBHA protein treatments

The recombinant HBHA protein was expressed and purified as previously described (18). RAW264.7 and ECSIT^KD^ RAW264.7, either subjected to starvation induction or infected with BCG (MOI = 10:1), were treated with HBHA at final concentrations of 5 μg/mL and 10 μg/mL. Autophagic activity was subsequently assessed to evaluate the inhibitory effects of HBHA-ECSIT interaction on macrophage autophagy.

### 2.4 Co-immunoprecipitation assay

RAW264.7 were infected or non-infected with BCG (MOI = 10:1) in the presence of varying concentrations (0-10 μg/mL) of HBHA for 48 hours. Cells were washed once with ice-cold 1× PBS and lysed with RIPA lysis buffer (containing 1 mM PMSF). After 10 min incubation on ice, complete cell disruption was achieved through repetitive pipetting. Lysates were centrifuged at 14,000 × g for 10 min at 4 °C, and supernatants were collected for following steps. Protein A magnetic beads were resuspended in lysis buffer, vortexed, and subjected to magnetic separation (15 sec) until clear supernatant was achieved. Cleared lysates (200 μL per sample) were incubated with anti-ECSIT polyclonal antibody (1:100 dilution) under continuous rotation at 4 °C overnight. Antigen-antibody complexes were then captured by adding 20 μL pre-washed magnetic beads, followed by 20 min rotary incubation at room temperature. Bead-bound complexes underwent five stringent washes with 500 μL ice-cold lysis buffer. Immunoprecipitates were eluted in 20-40 μL 4×Loading buffer through 10 min boiling at 95-100 °C. After magnetic separation, eluates were collected for subsequent western blot analysis.

### 2.5 Western blot analysis

Protein lysates were quantified via BCA assay and equal amounts were resolved on 12% SDS-PAGE gels. Electrophoretically separated proteins were transferred onto 0.45 μm PVDF membranes (Merck Millipore, USA). Membranes were blocked with 5% (w/v) non-fat dry milk (BD Biosciences, USA) in TBST buffer for 1 hour at room temperature (RT), followed by incubation with primary antibodies overnight at 4 °C. Subsequently, membranes were probed with HRP-conjugated secondary antibodies for 2 hours at RT. Detection was performed using an ECL method.

### 2.6 Immunofluorescence assay

Cells were seeded onto sterile glass coverslips placed in 12-well plates and cultured until reaching 80% confluence. Following experimental treatments, cells were washed three times with PBS to remove residual medium. Cells were fixed with 4% paraformaldehyde (PFA) for 10 min at RT, followed by three PBS washes. Permeabilization was performed using 0.2% Triton X-100 in PBS for 10 min at RT, with subsequent PBS rinses. Non-specific binding was blocked with 1% bovine serum albumin (BSA) in PBS for 1 hour at RT. After three PBS washes, cells were incubated overnight at 4 °C with primary anti-LC3 antibody. Following three PBS washes, FITC-conjugated secondary antibodies were applied for 1–2 hour at RT under light-protected conditions. After final PBS washes, nuclei were counterstained with DAPI in PBS for 15 min at RT. Coverslips were mounted onto glass slides using 20 μL anti-fade mounting medium and imaged using a laser scanning confocal microscope (Olympus FV4000) with 63× oil immersion objective.

### 2.7 Survival of BCG in macrophages

RAW264.7 and ECSIT^KD^ RAW264.7 maintained in RPMI-1640 medium supplemented with 10% FBS were infected with BCG at MOI = 10:1 in the presence of varying concentrations of HBHA. Following different hours bacterial internalization, extracellular BCG was eliminated by 50 μg/mL gentamicin treatment for 1 h. Before lysis, the monolayer cells were gently washed twice with PBS to remove detached dead cells and residual extracellular debris. Washed monolayer cells were lysed with 0.02% SDS for 15 min at 37°C. Lysates were centrifuged at 12,000 × g for 30 min. Pelleted bacilli were resuspended in PBS, serially diluted (10^-^² to 10^-4^), and plated on Middlebrook 7H10 agar supplemented with OADC enrichment. Colonies were counted after 3 weeks incubation at 37 °C.

### 2.8 Statistical analysis

All values were expressed as mean ± SD. Data were analyzed by one-way ANOVA with Bonferroni correction for multiple comparisons. The follow-up least significant difference test was used for *post hoc* comparison with assess differences between groups. Differences with *p* values <0.05 considered to be statistically significant.

## 3 Results

### 3.1 HBHA disrupts ECSIT-TRAF6 interaction and inhibits ECSIT ubiquitination

**Figure 1:**
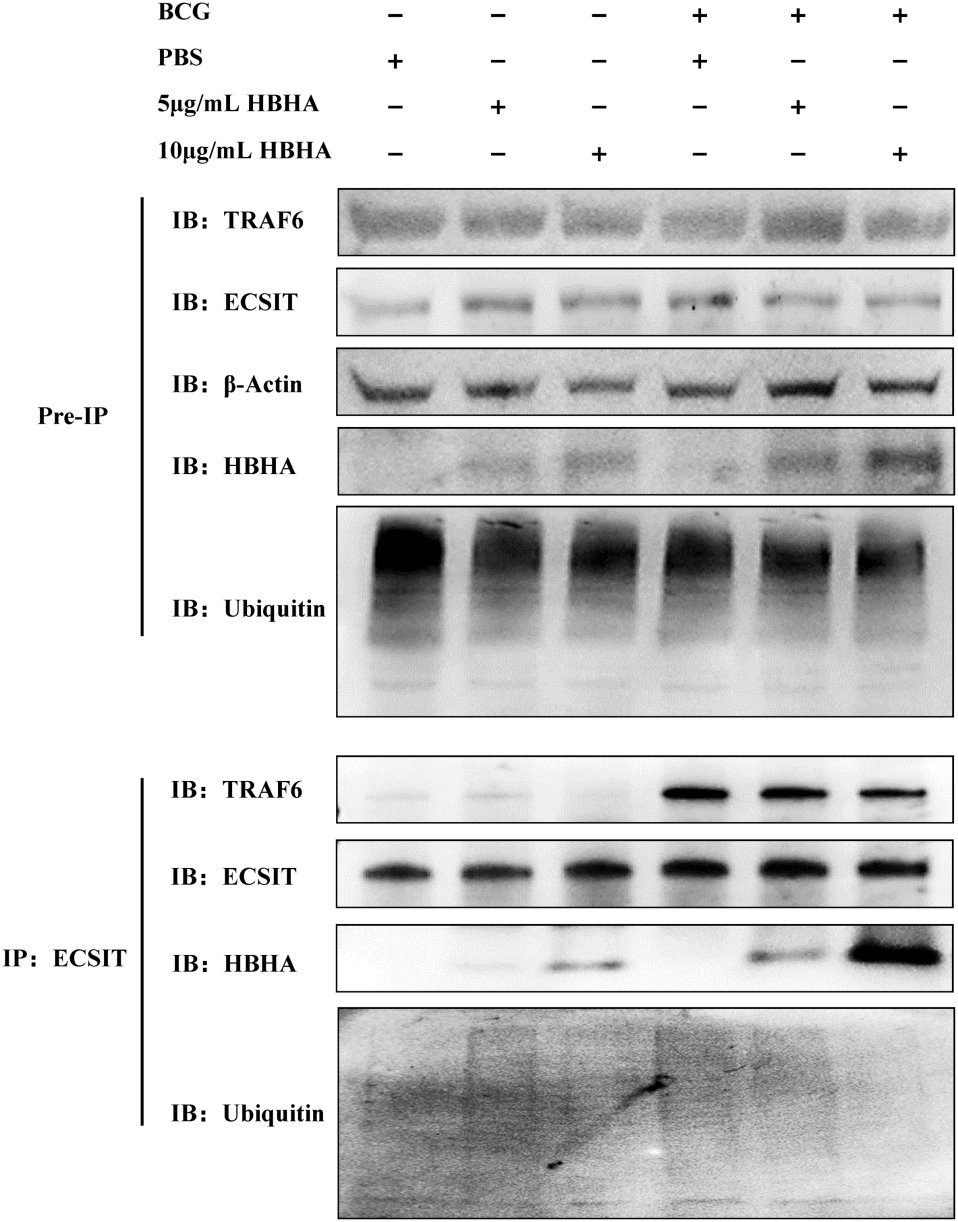
Heparin-binding hemagglutinin (HBHA) disrupts ECSIT-TRAF6 interaction and inhibits ECSIT ubiquitination. RAW264.7 cells were infected with BCG (MOI = 10:1) in the presence of varying concentrations (0, 5 or 10 μg/mL) of HBHA for 48 hours. Cell lysates were immunoprecipitated with anti-ECSIT antibody followed by immunoblotting with anti-TRAF6, anti-HBHA, anti-ECSIT or anti-Ubiquitin antibody.

Previous studies have established that the assembly of the TRAF6-ECSIT complex, which facilitates ECSIT ubiquitination, plays a critical role in TLR4-mediated NF-κB activation required for intracellular bacterial clearance in macrophages (22, 24). Building on our initial discovery of direct HBHA-ECSIT interaction through HuProt™ human proteome microarray screening, we performed co-IP assays in RAW264.7 to investigate whether HBHA binding modulates ECSIT-TRAF6 complex formation. RAW264.7 cells were infected with BCG (MOI = 10:1) in the presence of varying concentrations of HBHA for 48 hours. Subsequent immunoprecipitation with anti-ECSIT antibody revealed dose-dependent HBHA co-precipitation with ECSIT, particularly enhanced in BCG-infected groups (**Figure 1**). Conversely, TRAF6 interaction with ECSIT showed progressive reduction with increasing HBHA concentrations, demonstrating effective disruption of the TRAF6-ECSIT complex by HBHA binding. This disruption of interaction directly correlated with diminished ECSIT ubiquitination levels in macrophages (**Figure 1**).

Our findings establish a novel mechanism whereby mycobacterial HBHA subverts host defenses through direct interaction with ECSIT. This pathogen-host protein interaction effectively inhibits both ECSIT ubiquitination and subsequent downstream signaling pathways, representing the evidence of mycobacterial HBHA directly targeting the ECSIT complex during macrophage invasion.

### 3.2 HBHA inhibits autophagy in RAW264.7 through interaction with ECSIT

Macrophages play a pivotal role in host defense against Mtb infection (13). According to our prior discovery that HBHA inhibits macrophage autophagy (18), we investigated whether ECSIT mediates this suppression. RAW264.7 and ECSIT^KD^ RAW264.7 were exposed to varying concentrations of HBHA, and autophagic activity was assessed by monitoring LC3-II conversion (a hallmark of autophagosome formation) and Beclin-1 expression (a key regulator of autophagosome nucleation).

**Figure 2:**
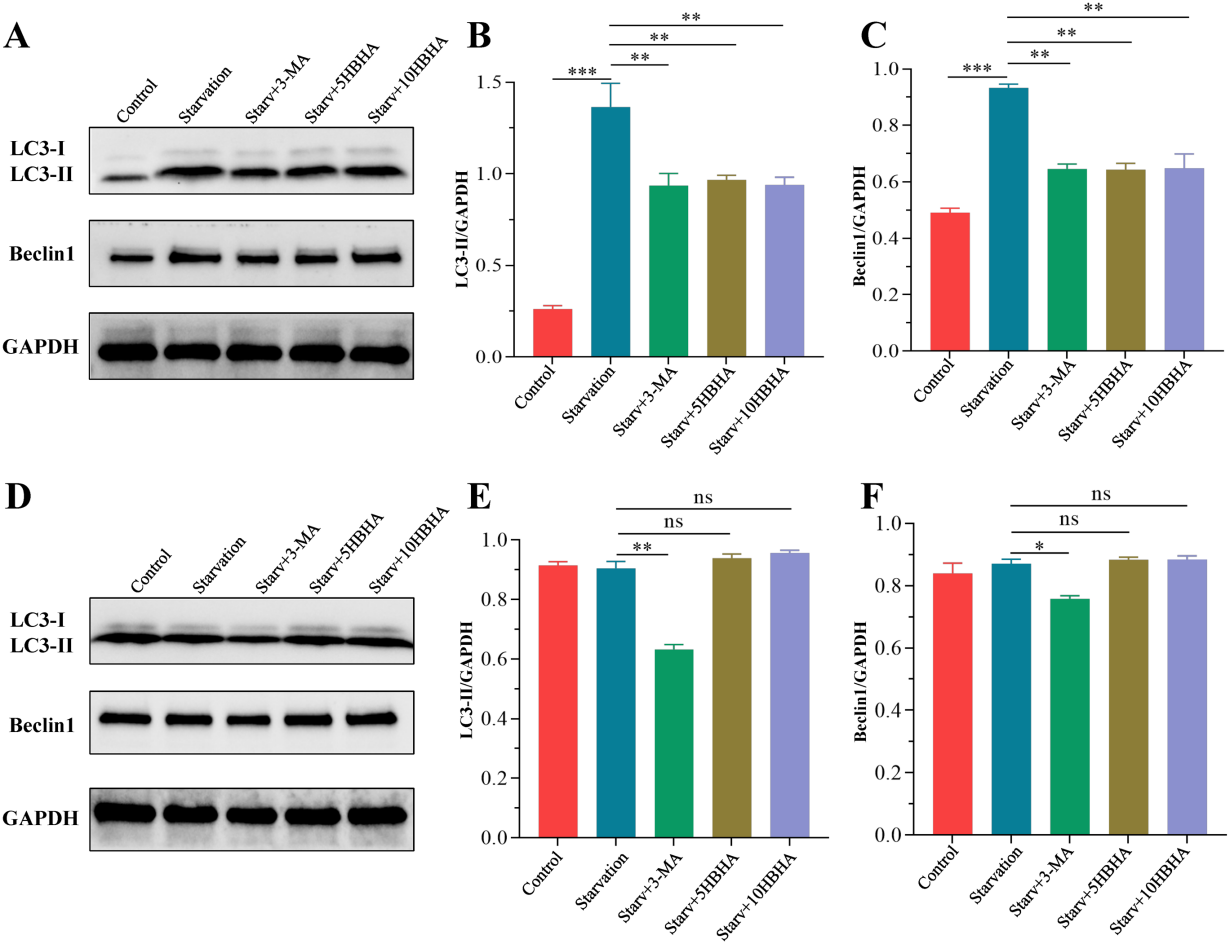
HBHA suppresses starvation-induced autophagy markers via ECSIT. RAW264.7 **(A)** and ECSIT^KD^ RAW264.7 **(D)** were starved for 4 hours, then treated with 100 μM 3-MA (autophagy inhibitor), 5 μg/mL HBHA or 10 μg/mL HBHA for 6 hours. Total cellular proteins were collected for detection of LC3 and Beclin1 expression. **(A)** Expression of LC3 and Beclin1 in RAW264.7 were detected by western blot. **(B, C)** The intensity of LC3-II and Beclin1 bands was normalized to the intensity of GAPDH. **(D)** Expression of LC3 and Beclin1 in ECSIT^KD^ RAW264.7 were detected by western blot. **(E, F)** Quantification of LC3-II/GAPDH and Beclin1/GAPDH ratios from the western blot analysis. Data shown are from a single representative experiment. Each value indicates mean ± SD of results obtained from three independent experiments. *p < 0.05, **p < 0.01, ***p < 0.001, ns, not significant.

In RAW264.7, starvation significantly increased LC3-II levels compared to untreated controls (p < 0.005), while the autophagy inhibitor 3-MA reduced LC3-II accumulation (p < 0.01). Strikingly, HBHA treatment significantly attenuated starvation-induced LC3-II elevation (**Figures 2A, B**). Moreover, starvation upregulated Beclin-1 expression, which was suppressed by both 3-MA and HBHA (**Figures 2A, C**). Intriguingly, genetic ablation of ECSIT abolished the autophagy-inhibitory effects of HBHA. In ECSIT^KD^ RAW264.7, starvation-induced LC3-II levels remained unchanged upon HBHA treatment, converse results observed with 3-MA (**Figures 2D, E**). Similarly, Beclin-1 expression in ECSIT^KD^ RAW264.7 exhibited no significant differences between starvation and HBHA-treated groups, whereas 3-MA retained its inhibitory effect (p < 0.01, **Figures 2D, F**).

**Figure 3:**
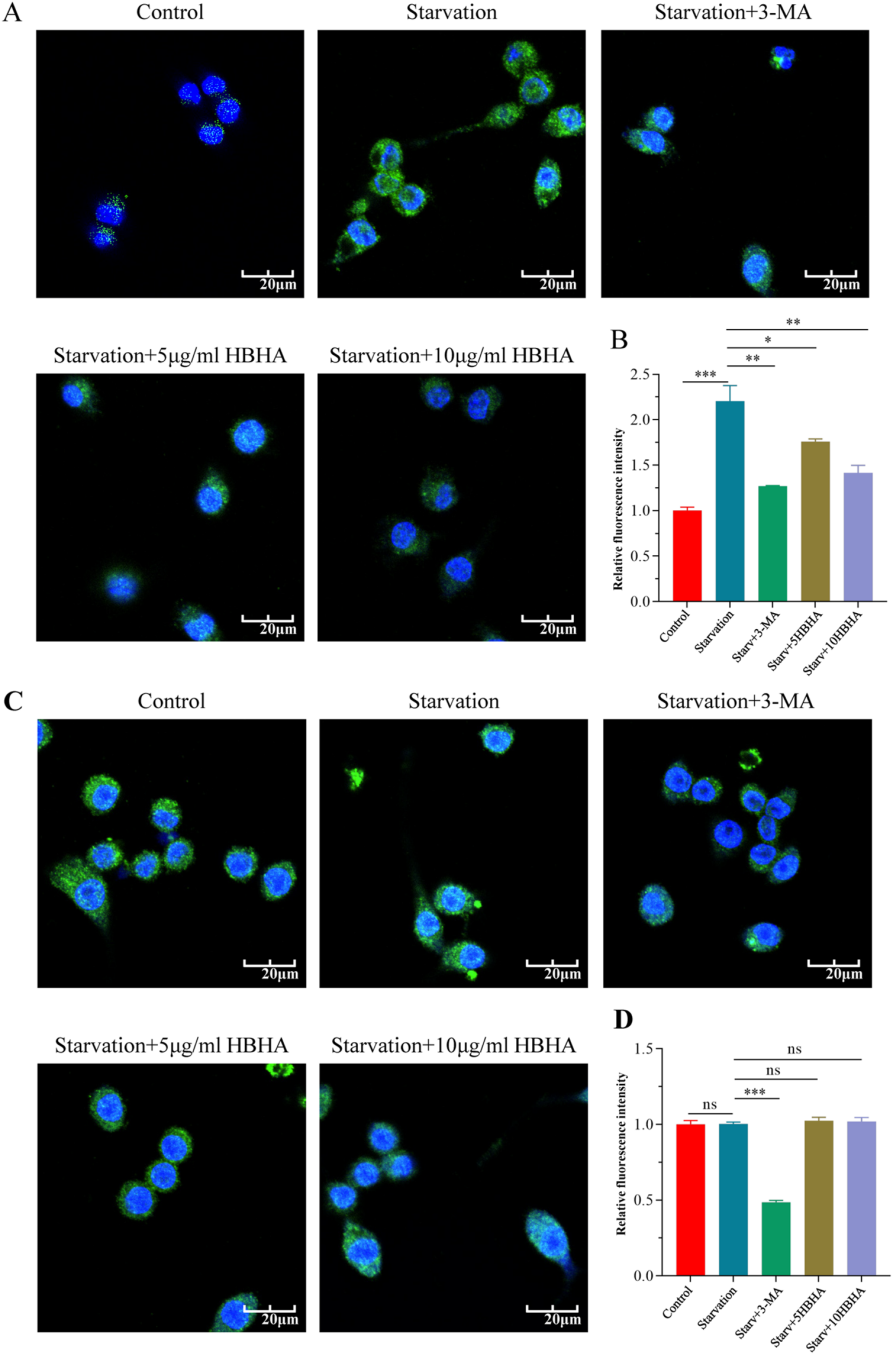
HBHA attenuates LC3 puncta formation in an ECSIT-dependent manner. RAW264.7 **(A)** and ECSIT^KD^ RAW264.7 **(C)** were starved for 4 hours and subsequently treated with 3-MA (100 μM) or HBHA (5 μg/mL/10 μg/mL) for 6 hours. Cells were then stained for nuclei (DAPI, blue) and LC3 (green). **(A)** Immunofluorescence of LC3 (green) in RAW264.7 was detected by confocal microscopy. Scale bars: 20 μm. **(B)** Quantification of LC3 puncta per cell in RAW264.7. **(C)** Immunofluorescence of LC3 (green) in ECSIT^KD^ RAW264.7 was detected by confocal microscopy. Scale bars: 20 μm. **(D)** Quantification of LC3 puncta per cell from the immunofluorescence analysis shown in **(C)**. Data shown are from a single representative experiment. Each value in **(B, D)** indicates mean ± SD of results obtained from three independent experiments. *p < 0.05, **p < 0.01, ***p < 0.001, ns, not significant.

To further validate the role of HBHA-ECSIT interaction in autophagy suppression, we observed autophagosome formation through immunofluorescence staining of LC3 in RAW264.7 and ECSIT^KD^ RAW264.7 (**Figure 3**). In RAW264.7, nutrient deprivation significantly elevated LC3 puncta formation compared to untreated controls (p < 0.005), which was attenuated by 3-MA treatment (p < 0.01), and HBHA dose-dependently suppressed starvation-induced autophagosome formation (**Figures 3A, B**). Remarkably, while 3-MA further reduced LC3 puncta in ECSIT^KD^ RAW264.7 (p < 0.005), HBHA treatment showed no inhibitory effect (**Figures 3C, D**).

Therefore, all these findings demonstrate that Mtb virulence factor HBHA specifically targets ECSIT to impair autophagic flux, revealing a molecular pathway that could be exploited for developing autophagy-modulating therapies against LTBI.

### 3.3 HBHA-ECSIT interaction subverts mycobacterial infection-induced autophagy in macrophages

**Figure 4:**
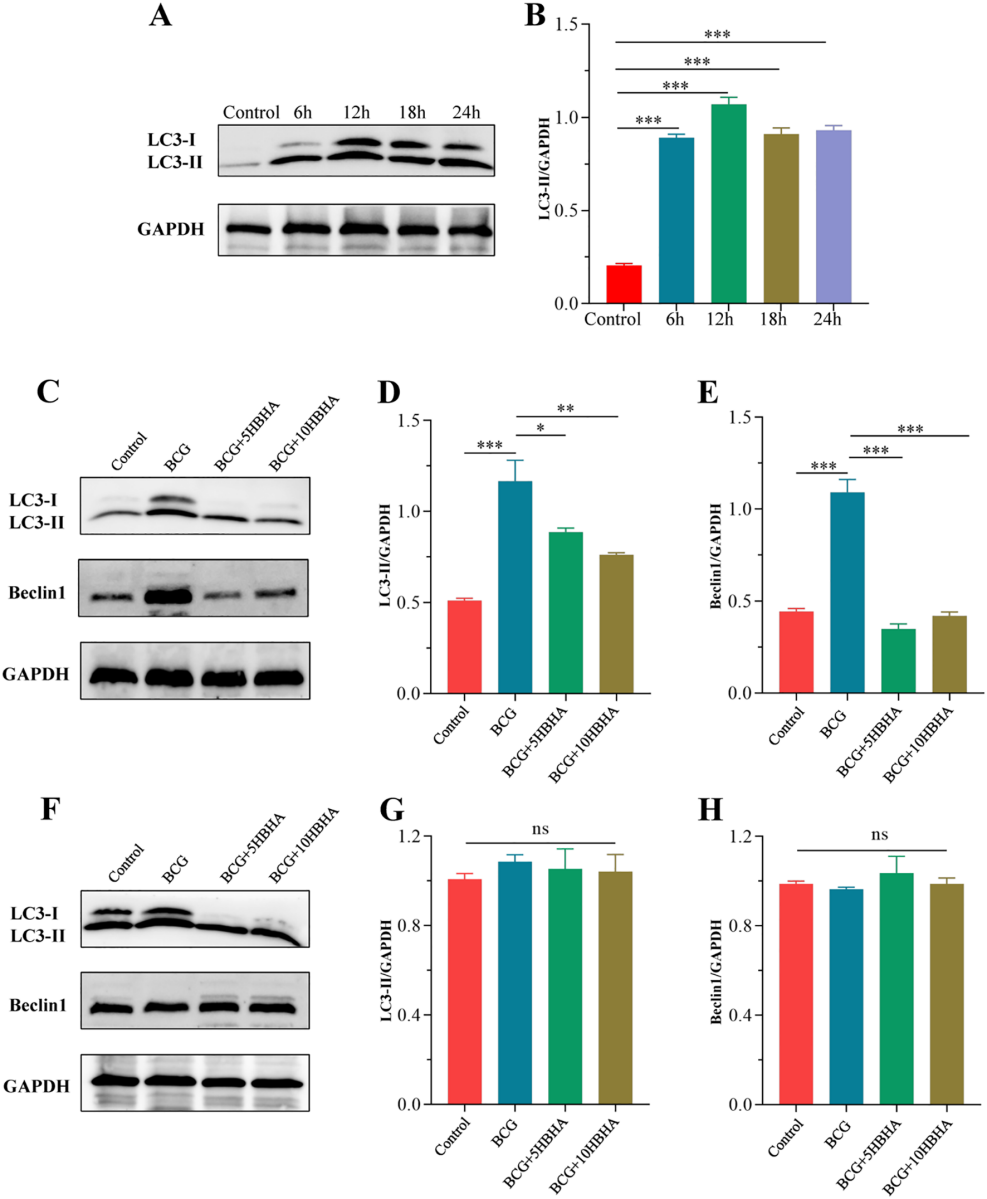
ECSIT is required for HBHA-mediated inhibition of BCG-induced autophagy. **(A)** RAW264.7 were infected with BCG at MOI = 10:1 for different times (6 hours, 12 hours, 18 hours or 24 hours). Cells were extracted and expression of LC3 was detected by western blot. **(B)** The intensity of LC3-II bands was normalized to the intensity of GAPDH. **(C-H)** RAW264.7 and ECSIT^KD^ RAW264.7 were infected with BCG at MOI = 10:1 and treated with HBHA (0, 5 or 10 μg/mL) for 12 hours. Cells were extracted and total cellular proteins were collected respectively. **(C)** Expression of LC3 and Beclin1 in RAW264.7 were detected by western blot. **(D, E)** Quantification of LC3-II/GAPDH and Beclin1/GAPDH ratios from the western blot analysis shown in **(C, F)** Expression of LC3 and Beclin1 in ECSIT^KD^ RAW264.7 were detected by western blot. **(G, H)** The intensity of LC3-II and Beclin1 bands was normalized to the intensity of GAPDH. Data shown are from a single representative experiment. Each value indicates mean ± SD of results obtained from three independent experiments. *p < 0.05, **p < 0.01, ***p < 0.001, ns, not significant.

To elucidate how HBHA-ECSIT interaction modulates macrophage autophagy during mycobacterial infection, we established a time-course infection model using *M. bovis* BCG (MOI = 10) in RAW264.7. Western blot analysis revealed time-dependent modulation of autophagy marker LC3-II in RAW264.7: Significant elevation compared to uninfected controls (p<0.01) at 6 h post-infection, peaking at 12 h (p<0.005), followed by gradual decline at 18/24 h while remaining above baseline (**Figures 4A, B**). This kinetic profile identified 12 h as the optimal infection duration for subsequent experiments. Following mechanistic investigations demonstrated that HBHA treatment significantly attenuated BCG-induced LC3-II and Beclin-1 expression compared to infection alone (**Figures 4C–E**). Strikingly, LC3-II/Beclin-1 expression in ECSIT^KD^ RAW264.7 were not significantly different (p>0.05) among uninfected, BCG-infected, or HBHA-treated groups (**Figures 4F–H**), suggesting that ECSIT deficiency may constitutively activate autophagic pathways and HBHA specifically inhibits BCG-triggered macrophage autophagy through ECSIT targeting.

**Figure 5:**
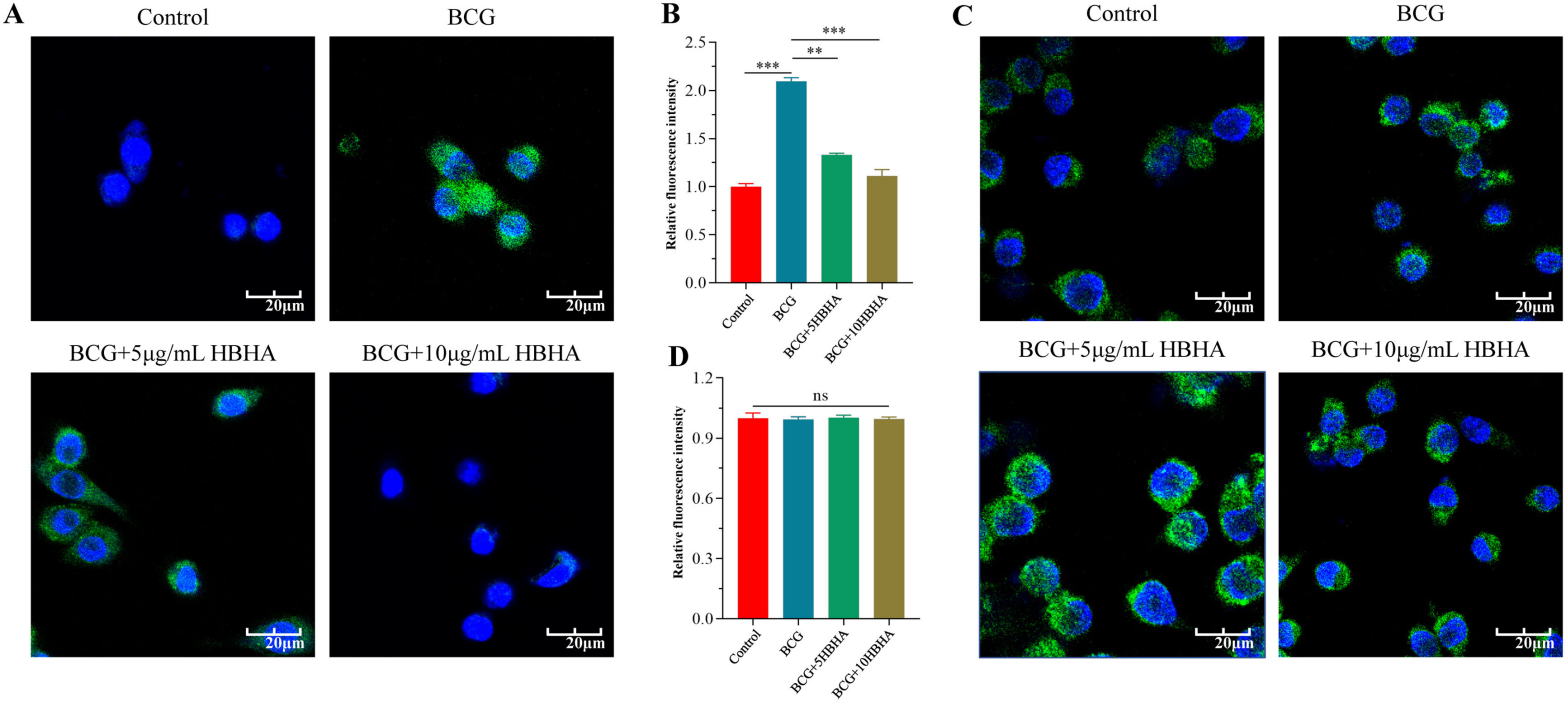
HBHA requires ECSIT to diminish LC3 aggregation during BCG infection. RAW264.7 and ECSIT^KD^ RAW264.7 were infected with BCG at MOI = 10:1 in the presence of HBHA (0, 5 or 10 μg/mL) for 12 hours. Cells were then stained for nuclei (DAPI, blue) and LC3 (green). **(A)** Immunofluorescence of LC3 (green) in RAW264.7 was detected by confocal microscopy. Scale bars: 20 μm. **(B)** Quantification of LC3 puncta per cell in RAW264.7. **(C)** Immunofluorescence analysis of LC3 puncta formation in ECSIT^KD^ RAW264.7. Scale bars: 20 μm. **(D)** Quantification of LC3 puncta per cell from the immunofluorescence analysis shown in **(C)**. Data shown are from a single representative experiment. Each value in **(B, D)** indicates mean ± SD of results obtained from three independent experiments. **p < 0.01, ***p < 0.001, ns, not significant.

Based on these observations, we performed immunofluorescence microscopy to visualize LC3 puncta formation in BCG-infected macrophages. In RAW264.7, co-localization analysis of DAPI-stained nuclei (blue) and FITC-labeled LC3 (green) revealed that BCG infection (MOI = 10, 12 h) significantly increased LC3-positive puncta compared to uninfected controls (p<0.005), whereas HBHA treatment reduced puncta density substantially versus infection alone (**Figures 5A, B**). Notably, there was no statistically significant difference (p>0.05) in LC3 fluorescence intensity among uninfected, BCG-infected, or HBHA-treated groups in ECSIT_KD_ RAW264.7 (**Figures 5C, D**), which consistent with western blot results.

Collectively, these findings provide conclusive evidence that HBHA exploits ECSIT to subvert mycobacterial infection-induced autophagy, and this regulatory axis strictly dependent on intact ECSIT expression, which reveals a novel immune evasion strategy employed by mycobacteria.

### 3.4 Inhibition of autophagy by HBHA-ECSIT interaction promotes Mycobacterium survival in macrophages

To investigate the functional consequences of autophagy suppression by HBHA-ECSIT interaction on mycobacterial persistence, we performed intracellular bacterial survival assays in RAW264.7 and ECSIT^KD^ RAW264.7. Cells were infected with BCG at a MOI of 10 in the presence of varying concentrations (0-10 μg/mL) of HBHA. CFU enumeration was performed at 6, 24, and 48 h post-treatment through serial dilution plating on 7H10 Middlebrook agar.

**Figure 6:**
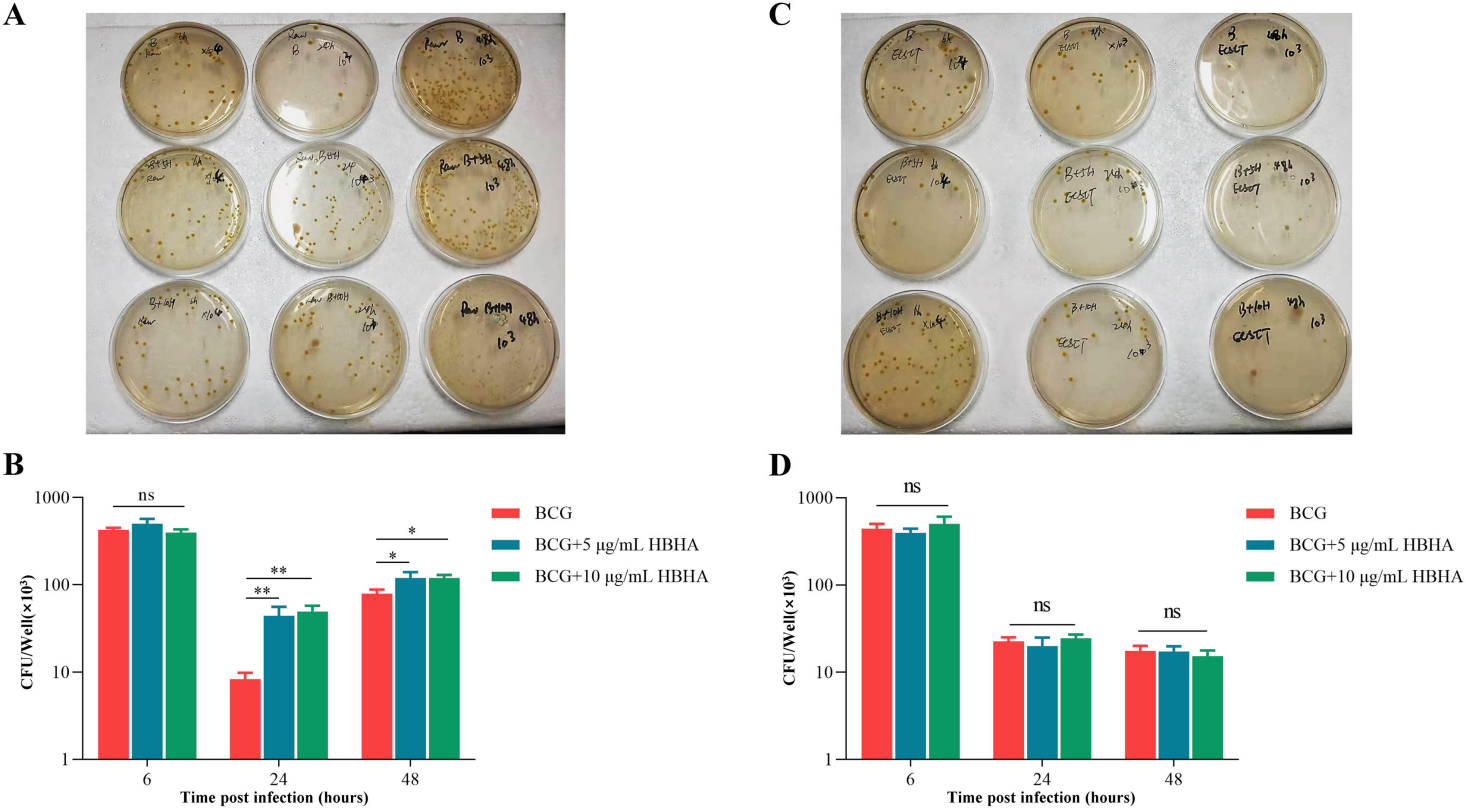
Inhibition of autophagy by HBHA-ECSIT interaction promotes BCG persistence in macrophages. RAW264.7 and ECSIT^KD^ RAW264.7 were infected with BCG at MOI = 10:1 and treated with HBHA (0, 5 or 10 μg/mL) for 6, 24 or 48 hours. Cells were lysed with 0.02% SDS for 15 min at 37 °C. The lysates were diluted and plated on 7H10 agar plates to count the number of intracellular bacteria. **(A)** The lysates of BCG-infected RAW264.7 were plated on 7H10 agar plates. **(B)** Colonies was counted to obtain the number of intracellular bacteria in RAW264.7. **(C)** The lysates of BCG-infected ECSIT^KD^ RAW264.7 were plated on 7H10 agar plates. **(D)** Quantification of Colony-forming unit (CFU) from the analysis shown in **(C)**. Data shown are from a single representative experiment. Each value in **(B, D)** indicates mean ± SD of results obtained from three independent experiments. *p < 0.05, **p < 0.01, ns, not significant.

As shown in **Figures 6A, B**, initial infection efficiency analysis revealed comparable intracellular BCG loads across all groups at 6 h post-infection, confirming that HBHA does not influence bacterial adherence or invasion into macrophages. By 24 h, while overall CFU counts decreased in all groups due to experimental clearance of non-viable cells, HBHA-treated macrophages retained significantly higher bacterial loads compared to untreated controls. This survival advantage still was pronounced at 48 h, with HBHA-treated macrophages sustaining higher CFUs than untreated controls. However, genetic ablation of ECSIT completely abrogated HBHA’s pro-survival effects (**Figures 6C, D**). No significant differences in CFU counts were observed between HBHA-treated and untreated groups at any timepoint, demonstrating absolute ECSIT-dependence of HBHA-mediated bacterial persistence through autophagy suppression. The loss of HBHA-mediated bacterial persistence in ECSIT^KD^ macrophages directly correlates with our earlier findings of restored autophagic flux, confirming that ECSIT is indispensable for HBHA’s autophagy-subverting activity.

## 4 Discussion

Mtb is a highly adapted intracellular pathogen that causes tuberculosis (1, 11). Macrophage autophagy appears to play critical roles in defending against Mtb infection, by preventing pathogens from exploiting host cells for growth and multiplication. Once Mtb are internalized, macrophages would initiate the autophagy process for intracellular Mtb clearance, confirming the protective role of autophagy in host defense against Mtb. However, Mtb employs multiple mechanisms to evade autophagic clearance (12–15). Our previous research identified that Mtb virulence factor HBHA potently inhibits macrophage autophagy to facilitate bacterial survival (18), yet its molecular mechanisms remain elusive.

As a critical virulence factor of Mtb, HBHA not only mediates bacterial adhesion to epithelial cells and extrapulmonary dissemination but also plays specific roles in latent infection (19, 25, 26). Previous studies have established that HBHA, highly expressed during Mtb dormancy, selectively stimulates IFN-γ secretion in CD4^+^ T cells from individuals with LTBI, making it a diagnostic biomarker for LTBI (26–28). Our prior work further demonstrated that HBHA suppresses autophagosome formation in A549 epithelial cells while activating caspase-3-dependent apoptosis (29). Notably, its autophagy-inhibitory effects in macrophages strongly implicate HBHA as a central player in tuberculosis latency (18), though the underlying mechanisms remained elusive.

Through HuProt™ human proteome microarray screening, we identified a novel interaction between HBHA and ECSIT, a multifunctional protein in innate immunity. ECSIT orchestrates TLR/NF-κB signaling via TRAF6-mediated ubiquitination and serves as a Parkin substrate during mitophagy (21, 22). Co-IP assays confirmed that HBHA directly binds ECSIT during Mtb infection, disrupting the ECSIT-TRAF6 complex and inhibiting ECSIT ubiquitination (**Figure 1**). This represents the first direct evidence of interaction between mycobacterial HBHA and host ECSIT during macrophages invaded by Mtb. Notably, this binding was maximal in BCG-infected macrophages, likely due to synergy between endogenous HBHA expressed by BCG and exogenous recombinant HBHA, combined with BCG infection-induced ECSIT upregulation or relocalization via innate immune pathways (30–32).

To delineate ECSIT’s central role in HBHA-mediated autophagy suppression, we exploited ECSIT-knockdown RAW264.7 macrophages. Western blot and immunofluorescence analyses revealed that HBHA dose-dependently inhibited LC3-II conversion and Beclin-1 expression in RAW264.7, effects completely abolished in ECSIT^KD^ RAW264.7 (**Figures 2**–**4**, **6**). Intriguingly, ECSIT^KD^ RAW264.7 exhibited elevated basal autophagy, with neither starvation nor BCG infection further augmenting autophagic flux (**Figures 2D**, **3C**, **4F**, **5C**). This suggested dual regulatory roles of ECSIT, as evidenced by prior work (21–23): 1) maintaining basal autophagy homeostasis via Parkin-dependent mitophagy, and 2) mediating infection-induced autophagy through TRAF6 ubiquitination. ECSIT ablation likely disrupts mitochondrial quality control, leading to constitutive autophagy activation. Importantly, in ECSIT^KD^ RAW264.7, 3-MA retained its inhibitory effects by targeting PI3K in a manner independent of ECSIT, whereas HBHA lost efficacy due to the absence of its molecular target.

Using a BCG infection model, we further demonstrated that HBHA significantly enhanced intracellular BCG survival in RAW264.7 (**Figures 6A, B**), an effect entirely abrogated in ECSIT^KD^ RAW264.7 (**Figures 6C, D**). These findings established ECSIT as the essential molecular nexus through which HBHA subverts autophagy to promote bacterial persistence, indicating that HBHA-ECSIT interaction exerted pathogenic effects of blocking autophagolysosomal clearance.

While our study utilized BCG infection with exogenously added recombinant HBHA to investigate the HBHA-ECSIT interaction under controlled conditions, we recognize that this model may not fully recapitulate HBHA dynamics during natural Mtb infection, especially as BCG lacks the ESX-1 system, which enables Mtb phagosomal permeabilization and effector release (e.g., HBHA) into the cytosol for ECSIT interaction (33–35). Our exogenous HBHA approach thus simulates this access. The recombinant HBHA concentrations were chosen based on our and other previous studies showing biological activity in similar experimental systems (18, 29), though their physiological relevance in infected macrophages requires confirmation. Nonetheless, translational potential is supported by HBHA’s conservation across Mtb isolates and ECSIT’s evolutionary preservation, implying similar axis function in Mtb where ESX-1 exposes ECSIT to secreted HBHA (30, 33, 34). Future work with HBHA-deficient H37Rv strains in primary macrophages and *in vivo* models will validate pathophysiological roles and guide therapies for persistent infections.

This study provides the first mechanistic evidence that HBHA, an LTBI-associated antigen, inhibits macrophage autophagy via ECSIT targeting, offering critical insights into early immune evasion during latent infection and highlighting HBHA-ECSIT axis as a potential host-directed therapeutic target.

## Data Availability

The original contributions presented in the study are included in the article/supplementary material. Further inquiries can be directed to the corresponding author.
